# SEVA 4.0: an update of the Standard European Vector Architecture database for advanced analysis and programming of bacterial phenotypes

**DOI:** 10.1093/nar/gkac1059

**Published:** 2022-11-24

**Authors:** Esteban Martínez-García, Sofía Fraile, Elena Algar, Tomás Aparicio, Elena Velázquez, Belén Calles, Huseyin Tas, Blas Blázquez, Bruno Martín, Clara Prieto, Lucas Sánchez-Sampedro, Morten H H Nørholm, Daniel C Volke, Nicolas T Wirth, Pavel Dvořák, Lorea Alejaldre, Lewis Grozinger, Matthew Crowther, Angel Goñi-Moreno, Pablo I Nikel, Juan Nogales, Víctor de Lorenzo

**Affiliations:** Systems Biology Department, Centro Nacional de Biotecnología (CNB-CSIC), 28049 Cantoblanco-Madrid, Spain; Systems Biology Department, Centro Nacional de Biotecnología (CNB-CSIC), 28049 Cantoblanco-Madrid, Spain; Systems Biology Department, Centro Nacional de Biotecnología (CNB-CSIC), 28049 Cantoblanco-Madrid, Spain; Systems Biology Department, Centro Nacional de Biotecnología (CNB-CSIC), 28049 Cantoblanco-Madrid, Spain; Systems Biology Department, Centro Nacional de Biotecnología (CNB-CSIC), 28049 Cantoblanco-Madrid, Spain; Systems Biology Department, Centro Nacional de Biotecnología (CNB-CSIC), 28049 Cantoblanco-Madrid, Spain; Systems Biology Department, Centro Nacional de Biotecnología (CNB-CSIC), 28049 Cantoblanco-Madrid, Spain; Systems Biology Department, Centro Nacional de Biotecnología (CNB-CSIC), 28049 Cantoblanco-Madrid, Spain; Scienseed SL, 28020 Madrid,Spain; Scienseed SL, 28020 Madrid,Spain; Scienseed SL, 28020 Madrid,Spain; The Novo Nordisk Foundation Center for Biosustainability, Technical University of Denmark, 2800 Kongens Lyngby, Denmark; The Novo Nordisk Foundation Center for Biosustainability, Technical University of Denmark, 2800 Kongens Lyngby, Denmark; The Novo Nordisk Foundation Center for Biosustainability, Technical University of Denmark, 2800 Kongens Lyngby, Denmark; Department of Experimental Biology, Faculty of Science, Masaryk University, Brno 62500 Czech Republic; Centro de Biotecnología y Genómica de Plantas, Universidad Politécnica de Madrid (INIA-CSIC), Pozuelo de Alarcón 28223, Spain; Centro de Biotecnología y Genómica de Plantas, Universidad Politécnica de Madrid (INIA-CSIC), Pozuelo de Alarcón 28223, Spain; School of Computing, Newcastle University, NE4 5TG, UK; Centro de Biotecnología y Genómica de Plantas, Universidad Politécnica de Madrid (INIA-CSIC), Pozuelo de Alarcón 28223, Spain; School of Computing, Newcastle University, NE4 5TG, UK; Centro de Biotecnología y Genómica de Plantas, Universidad Politécnica de Madrid (INIA-CSIC), Pozuelo de Alarcón 28223, Spain; The Novo Nordisk Foundation Center for Biosustainability, Technical University of Denmark, 2800 Kongens Lyngby, Denmark; Systems Biology Department, Centro Nacional de Biotecnología (CNB-CSIC), 28049 Cantoblanco-Madrid, Spain; Systems Biology Department, Centro Nacional de Biotecnología (CNB-CSIC), 28049 Cantoblanco-Madrid, Spain

## Abstract

The SEVA platform (https://seva-plasmids.com) was launched one decade ago, both as a database (DB) and as a physical repository of plasmid vectors for genetic analysis and engineering of Gram-negative bacteria with a structure and nomenclature that follows a strict, fixed architecture of functional DNA segments. While the current update keeps the basic features of earlier versions, the platform has been upgraded not only with many more ready-to-use plasmids but also with features that expand the range of target species, harmonize DNA assembly methods and enable new applications. In particular, SEVA 4.0 includes (i) a sub-collection of plasmids for easing the composition of multiple DNA segments with MoClo/Golden Gate technology, (ii) vectors for Gram-positive bacteria and yeast and [iii] off-the-shelf constructs with built-in functionalities. A growing collection of plasmids that capture part of the standard—but not its entirety—has been compiled also into the DB and repository as a separate corpus (SEVAsib) because of its value as a resource for constructing and deploying phenotypes of interest. Maintenance and curation of the DB were accompanied by dedicated diffusion and communication channels that make the SEVA platform a popular resource for genetic analyses, genome editing and bioengineering of a large number of microorganisms.

## INTRODUCTION

At the onset of the recombinant DNA era, different initiatives were put forward to standardize the architecture and the nomenclature of plasmid vectors (1). In retrospect, that most—if not all—failed cannot come as a surprise, as all the benefits of standardization in terms of interoperability and reproducibility come at the cost of losing flexibility (2), an issue hardly acceptable by the time modern genetic engineering was being shaped. Yet, the development of synthetic biology (SynBio) decades later and its ambition to make biology easier to engineer has raised again the issue of standardization as one of the challenges that practitioners must seriously consider (3) and tackle for moving the field forward (4). A recent review has taken stock of where standardization lays in the contemporary landscape of SynBio-fuelled bioengineering and which are the low-hanging fruits that have been seized—or can be delivered in the near future (2). Among them stands the growing collection of molecular tools for both genetic analysis and phenotype design of bacteria beyond typical Laboratory workhorses like *Escherichia coli* and *Bacillus subtilis*. Such tools include on one hand formatted methods of DNA assembly (pioneered by the BioBrick concept; 5,6) and on the other hand, vectors for the deployment of given constructs in particular hosts (7–9).

**Figure 1.**
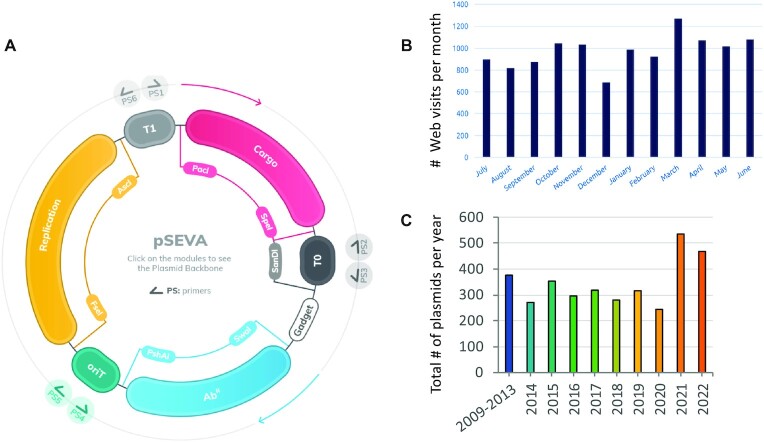
General organization of the SEVA plasmids and access statistics. (**A**) General organization of SEVA vectors as shown in the SEVA 4.0 homepage. All plasmids contain three basic modules (antibiotic marker, replication origin and cargo), the boundaries between being punctuated by unusual restriction sites. These are accompanied by a primary gadget site (additional sites are available also to this end). Clicking each of the segments enables users to visualize all choices and copy the cognate DNA sequence. The colored arrows above the cargo and Ab^R^ modules represent the directionality of the transcription flow of that element. (**B**) Visits to https://seva-plasmids.com during the period July 2021–June 2022. (**C**) Plasmids distributed since the onset of the SEVA platform.

It is in this context that the first SEVA database was launched in 2013 (10) with the purpose of bringing conceptual and material SynBio assets, in particular plasmid vectors, for analysis and construction of complex phenotypes to a much wider variety of bacteria. The proposed standard, (which has remained identical over subsequent versions) involves a few simple assembly rules of the functional DNA segments of the vectors i.e. replication origin, antibiotic marker and cargo. These segments are combined with a default *oriT* sequence for optional conjugal mobilization from the assembly host (typically *E. coli*) to the ultimate recipient (Figure 1A). The vectors resulting from such an arrangement are then assigned a code that acts as a unique identifier and since version SEVA 2.0 (11), they are also linked to a complete description in Synthetic Biology Open Language (SBOL) format (12). The community of users has grown ever since (Figure 1B) and the top 10 requested vectors have been distributed to hundreds of users (total plasmids distributed thus far exceeds 3400; Figure 1C) and the DB cited in the scientific literature >700 times at this time.

Since the latest update (13), the Open Access policy of the SEVA platform and its bottom-up, community-driven development have generated a substantial and useful feedback that is at the basis of the database update presented below. SEVA 4.0 incorporates a significant number of changes, modifications and improvements that are briefly described in the following paragraphs and fully reflected in the current webpage. Special efforts have been made to render SEVA vectors compatible with DNA assembly methods based on Type-II restriction enzymes as well as easing the interplay of users with more friendly interfaces for guiding vector selection and plasmid construction. On a different side, the growing availability of *in silico* analysis tools of DNA sequences and plasmid display software has advised to encourage users outsourcing such assets rather than having them embedded in the platform. Finally, we present below how the diffusion through social media channels attached to the DB has created a conspicuous, growing community of global users. Ultimately, the ambition of SEVA is to serve the Synthetic Biology community by offering an open and user-friendly collection of genetic tools that meets a whole range of needs from simple students' projects all the way to large-scale biotechnological applications.

## DATABASE DESCRIPTION

### Database organization

As was the case with its predecessor, the updated SEVA database (SEVA-DB 4.0, http://seva-plasmids.com/) serves primarily as an annotated and information-rich index of functional DNA sequences and constructs that are available to the community. The platform maintains a simple architecture, which consists of a relational database as the records storage layer, a series of modules and utilities that are hosted by an application server and a web-based presentation layer (13). The last is endowed with an explicit set of standards and keywords that apply to all constructs. The outputs of the search for each vector include links to GenBank (or alternatively to interim *gbk* or *gb* file of the plasmid), and to the sequences in Synthetic Biology Open language (SBOL) format (see below). However, built-in visualization of physical maps of each of the plasmids (which was available in earlier DB versions) has been removed and users are encouraged instead to export the DNA sequences of interest to exterior platforms of their choice, e.g. DNASTAR Lasergene (https://www.dnastar.com/) or Benchling (https://www.benchling.com/).

The graphic identity of the new website and its main sections have been largely kept as in the previous version (13), although it incorporates some important subsections and utilities that were not available before. The *Structure* section provides users with a basic description of the SEVA format as well as information about the steps needed for researchers to contribute to the standard—these have not changed significantly. The rules that govern the structure of the canonical SEVA plasmid collection are the very same proposed in 2013 (10) and we make a point of keeping them permanently as such. In this way, earlier vectors do not become obsolete and, instead, improvements can be entered by building on existing constructs. Such a canon involves DNA segments encoding specific plasmid-related functions which have been either synthesized or edited to remove restriction enzymes and endowed with rare restriction sites for assembly with an *oriT* sequence and transcriptional terminators in a fixed arrangement. Some of such rare sites can later be used for insertion of what we call *gadgets*, which endow some extra functionality to the vector (see below).

**Figure 2.**
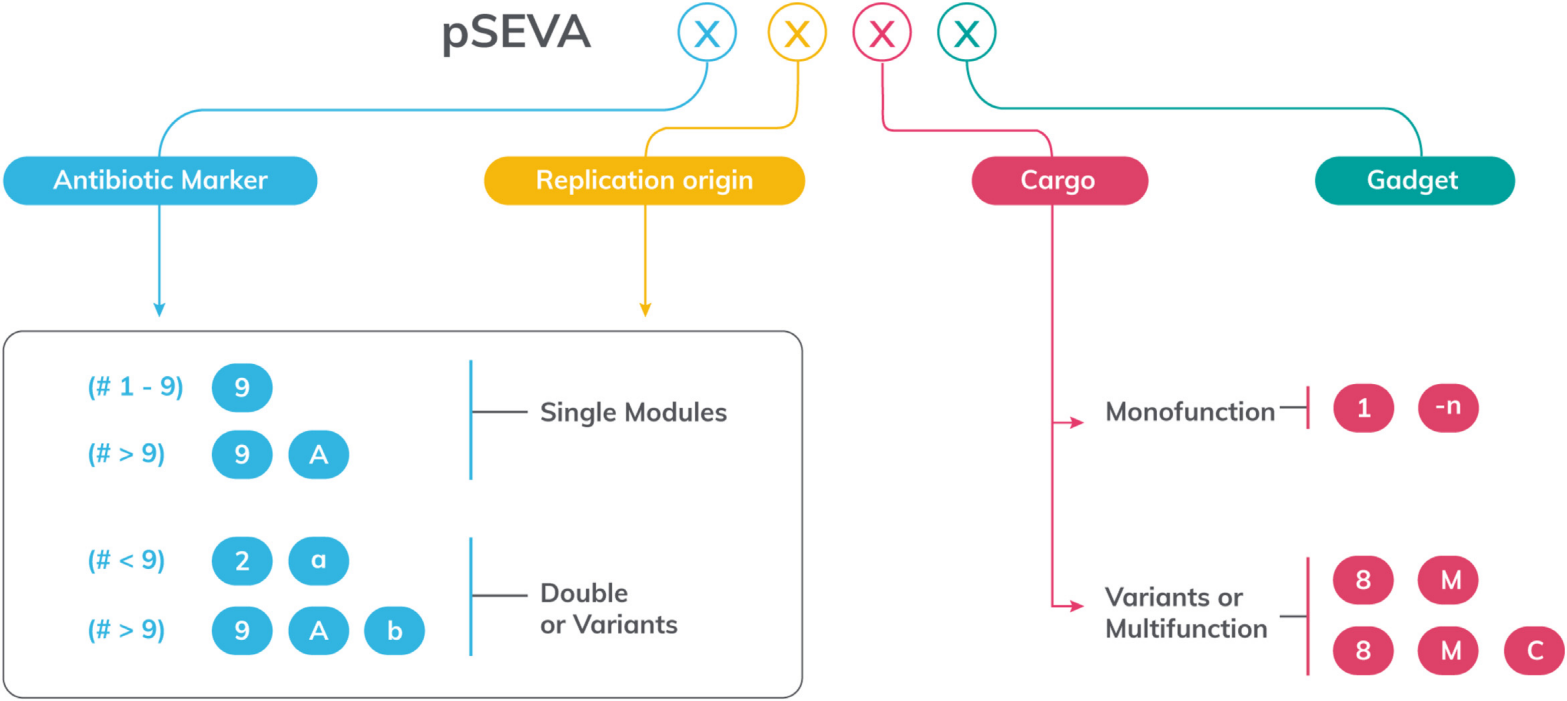
Updated nomenclature of SEVA vectors. The figure sketches the expanded rules for naming each of the four positions available for composing a complete SEVA code. The first position identifies the antibiotic resistance. The second position is the origin of replication (a sole numeric code 1 to 9 and then a capital letter is added). In case of either variants or addition(s) of either the antibiotic marker or a second replication origin, a lower-case letter is then inserted next. The third position is the cargo, which can be mono-function (named 1 to n) or variants/multi-function thereof (number followed by a capital letter). Finally, the fourth position is for the gadgets, which are designated by lowercase Greek letters (α to ω). For instance; pSEVA237M means a Km^R^ plasmid with a pBBR1 origin of replication and a promoterless *msf*GFP reporter (mono-function). pSEVA2313R means Km^R^, pBBR1 origin and a P_EM7_→mCherry cargo (bi-function). pSEVA2a2b8Rα means Km^R^ plasmid selectable in Gram-positive hosts with a double RK2-SCP2* replication origin, a bi-functional cargo *xylS*-P_m_→ mCherry and a *hok*-*sok* gadget (see text for explanation)

The *Backbone modules* section constitutes the core of the platform, as the user has access to an intuitive graphic tour through the different functional segments of the plasmids and where visitors can see and copy all relevant sequences of the SEVA frame (Figure 1A). This section also contains a graphic representation of the rules for naming the plasmids that follow the SEVA standard and the procedure to assemble its code. The members of the canonical collection are named with a non-ambiguous code which due to the growing number of entries, was revised in the current version as indicated in Figure 2 to describe e.g. variants of existing functional segments as well as a DNA fragment with two or more functions (see below).

**Figure 3.**
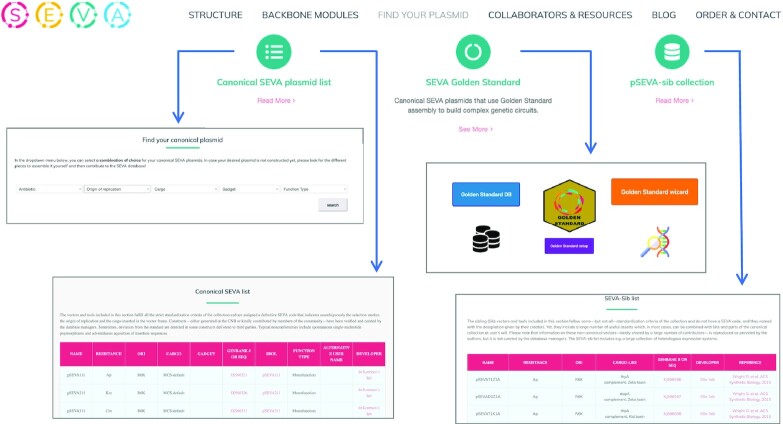
Details and utilities of the *Find you plasmid* section. The core of the database embodies the three subsections listed on top. Clicking each of them leads users to a different utility, the most useful of them being the Find your canonical plasmid, which can be composed according to necessities and then searched through the plasmid collection. The same section also leads to the resource named SEVA Golden Standard, which guides users through a complete roadmap for assembling complex constructs with Type IIS restriction technology. Separate Tables with the lists of canonical and non-canonical (SEVAsib) vectors can be accessed as well.

The next section (*Find your plasmid*; Figure 3) is the one which has been more significantly improved and offers more choices for users. The key subsection is named *Find your canonical plasmid*, where visitors can now separately select among many more antibiotic markers, replication origins and cargoes (instead of the earlier obligation to proceed stepwise). Optional gadgets include stabilization parts for securing plasmid retention in the absence of antibiotic selection and a yeast origin of replication for inter-kingdom transfer. Finally, users can survey a limited number of preset off-the-shelf functionalities for some specific applications (see below). This canonical collection (which can be perused also as a separate Table) includes the catalogue of SEVA vectors immediately available which fulfil every rule of the standard. While most plasmids of interest use to be ready in such a normative repository, combinations of functional parts that may not be physically assembled yet is facilitated to users-to-be by providing precursors following the directions spelled out in this same section. Availability of sequences in Genbank and SBOL formats enable their export for multiple visualisation options as indicated above.

### Highlights of the updated canonical SEVA collection

The most conspicuous addition to the ready-to-use, canonical vector list is the whole collection of plasmids grouped under the heading *SEVA Golden standard* (cargo #19 and derivatives). These plasmids enable a Type IIS assembly method for combinatorial multi-part composition of standardized genetic elements to create complex genetic constructs with many transcription units. The detailed description of such platform is described in (14) and this section of the SEVA webpage is linked to a distinct repository of composable parts and vectors along with tools for guiding the design of DNA segments with both stored and user-specific sequences.

Apart from the *SEVA Golden standard*, the updated DB and repository has been value-added with new and noteworthy cloning and expression vectors as well as pre-made constructs. These can immediately be used for particular purposes of ample interest regarding analyses and construction of bacterial phenotypes. First comes new SEVA-formatted cargoes e.g. (i) mTurquoise2 (in plasmid pSEVA2313T) where the gene encoding this marker was placed under the control of the constitutive P_EM7_ promoter (15), (ii) an intracellular pH sensor (assembled in plasmid pSEVA2513P), designed for assessing this parameter in Gram-negative bacteria. The cognate bi-functional cargo encodes a pH-sensitive reporter expressed through P_EM7_ (16), [iii] expanded tools for genome editing e.g. pSEVA1213S and pSEVA6213S, which supply the I-SceI meganuclease used for gene deletion in Gram-negative bacteria (17) endowed with the P_EM7_ promoter and bearing either an Ap^R^ or Gm^R^ resistance gene for specific applications. [iv] heterologous expression systems, e.g. responsive to either phosphate starvation (cargo #17 and its derivatives; 18) or rhamnose (cargo #18) as well as variants of existing ones thereof e.g. pSEVA6311 (a Gm^R^ vector encoding the cyclohexanone-inducible ChnR/P*_chnB_* system; 19,20). New expression systems also include a suite of calibrated constitutive promoters (cargoes #20-24) as well as an orthogonal T7 promoter (cargo #25) for high-processivity transcription of heterologous genes in Gram-negative hosts (21) that conditionally express the T7 RNA polymerase gene (for instance, integrated in the chromosome; 22). In some plasmids (e.g. pSEVA2325M or pSEVA2425M) the T7 promoter drives expression of an *msf*GFP marker (23) for calibration of transcriptional activity in given host strains.

Still within the realm of fully-standardized plasmids it is worth to highlight pSEVA2a2d1, which bear a double origin of replication (24) that enables constructs to become established in Gram-positive hosts (25). This adds to the already existing branch of SEVA vectors that replicate both in Gram-negative bacteria and actinomycetes (26). Also worthy of note is the yeast/bacteria shuttle vector pSEVA222Sβ, endowed with a CEN6-ARS209-URA origin of replication-selection marker insert at the gadget site of the plasmid backbone. This vector has been instrumental for assembling very large constructs using *S. cerevisiae* as the host for recombination of multiple DNA fragments (27).

### Registered SEVA siblings

Visitors of the SEVA webpage can also find a link to the list of SEVA sibling (SEVAsib) constructs in the section *Find your plasmid*, next to the *SEVA Golden standard* sector (Figure 3). The vectors and tools included in this divide follow some—but not all—standardization criteria of the collection, they do not have a SEVA code, and they are named with the designation provided by their creators. Yet, they include a large number of useful assets which, in most cases, can be combined with bits and parts of the canonical collection at the user's will. Please note that information on these non-canonical vectors—kindly shared by a large number of contributors—is reproduced as provided by the authors, but it is not curated by the database managers. Yet, the SEVAsib materials are available through the SEVA platform under the same terms of the canonical collection. However, specific details on their utilization may have to be requested to the original authors. Under these criteria, a suite of vectors and constructs that minimally diverge in respect to the standard were also incorporated to the update of SEVAsib section whenever rigorous maintenance of the format was not considered essential for the main purpose underlying their creation. For instance, some Ap^R^ vectors of the SEVAsib list bear a variant of the *bla* gene that has not been edited to fulfil the canon, but it delivers a much better selection with lower antibiotic concentrations (28). Also, the options for Type IIS-restriction mediated DNA assembly include not only the above-mentioned SEVA Golden vector collection, but also assets for what has been described as the SEVA 3.1 platform (29). This comprises a library of vectors tailored for Golden Gate-based assembly that work in conjunction with a core set of standard primers. This enables interoperability of the SEVA and BioBrick formats with minimal cloning and design efforts. That the deviation from the canon involved some swapping of the order of restriction sites in the cognate vectors (the so-called pSEVAb series)—while keeping the rest of the SEVA architecture—was an acceptable payoff for the interoperability of the plasmid vector series with the BioBrick assembly.

Another remarkable set of constructs of the SEVAsib collection comprises a whole inverter package for automated genetic circuit design in Gram-negative bacteria. This was aimed at extending the host range of the CELLO platform initially designed only for *E. coli* (30) towards a variety of non-canonical laboratory hosts (31–33). Also, a separate vector pSEVA6511-GIIi was tailored for recombination-independent insertion of short DNA sequences with different purposes. One of its applications involves introduction of unique identifiers (i.e. genetic barcodes; 34,35) in permissive genomic sites of bacteria of industrial or environmental interest for the sake of strain safety and traceability (36).

Besides, the updated webpage includes plasmids which act as suicide deliverers of transposon vectors (37). These endow users with the possibility of easy cloning and subsequent random insertion of functional cargoes with different antibiotic-resistance markers. A set of such vectors are based on Tn*5* (38) while another set is built on the Mariner transposase system—which, instead of a bias towards GC-rich regions, targets TA sites (39). In this last case, the plasmids bore the hyperactive form of the mariner Himar1 transposase (MarC9; 40) as well as cognate inverted repeats with a site for the MmeI type IIs enzyme (41,42) that cuts ∼20 bp away from its recognition site. This design allows inference of the genomic context of the inserted cargo and simplifies creation and analysis of next-generation sequencing libraries. While none of the transposon-delivery vectors—whether Tn*5*-based or Mariner-based—follows the SEVA rules altogether, the compatibility and boundaries of cloning sites makes transfer of cargoes among platforms straightforward.

Finally, the homepage of the platform incorporates (i) updated links to the addresses of depositors along with a connection to the autonomous *Plasmid collection of Ghent University* (within the Belgian Co-ordinated Collection of Microorganisms), a useful resource for Synthetic Biology-based biotechnology, (ii) access to the collection of blog posts in social media (see below) and (iii) contact forms for users to request the plasmids free of charge.

### Updating the nomenclature of SEVA modules

As the physical number of modules within SEVA plasmids continues to expand, the nomenclature of the collection has to evolve as well. While SEVA 3.0 included a detailed description of how-to code variant and double origins of replication nothing was stated about the antibiotic module (13). To clarify this, the update (Figure 2) reflects the nomenclature of different module variants encoding the same antibiotic resistance. While new antibiotics beyond #9 would be represented by adding a capital letter (9A, 9B and so on), different DNA variants for the same antibiotic resistance would be coded by adding a lower-case letter (similarly to the ORI module). For example, the new antibiotic codes #2a (*aadD* gene from pUB110-*S. aureus*) and #3a (*cat* gene from pC194-*S. aureus*) correspond to resistance cassettes derived from Gram-positive bacteria, that allows cognate bacterial cells to grow in kanamycin or chloramphenicol respectively (43–45). Moreover, the update includes SEVA plasmids adapted to modular cloning (Golden Standard; 14) which, as indicated above, leverages Type IIS assembly. The specific nomenclature of these plasmids is explained in (14).

Another new feature included in the 4.0 update is the grouping of SEVA plasmids into two types, mono-function or multi-function, depending on the contents of the cargo module. To meet that change, a new column has been added to the canonical SEVA plasmid list in the webpage. The mono-function group includes cargos endowed with a unique and clear application (e.g. MCS). The four types of cargoes applications originally described in SEVA 1.0 (10) belong to the mono-function class. These applications include the most common genetic operations: cloning, genome editing, promoter-probe and heterologous DNA expression systems. However, in some cases two functional applications can be joined together within a cargo module (the whole DNA sequence being flanked by PacI and SpeI) to deliver a new function. This multi-function category is not limited to just two functional applications, but as many as necessary. However, all such DNA sequences within this multi-functional cargo must fulfil the SEVA standard requirements (10). Multi-function plasmids include, e.g. constitutive/inducible expression of a reporter gene, or inducible expression of the I-SceI enzyme. This new criterion has allowed us to transfer plasmids previously listed as siblings into the canonical collection and thus re-name them to meet the updated SEVA code. In order to ease searches, an additional column with the earlier plasmid name has also been included in the canonical Table where necessary. The presence of multi-functional cargoes in given vectors is thus noted by first stating the leading part (e.g. P_EM7_ would be #13) followed by one or two letters corresponding to the downstream component (e.g. cargo P_EM7_→ mCherry would be #13R; see legend to Figure 2 for some examples)

### SEVA sequences in SBOL format: an update

SEVA 2.0 (11) enabled translation of vectors into the Synthetic Biology Open Language (SBOL) format, a data standard for genetic circuit design, the advantages of which have been widely argued before (12). In the current update, SEVAhub (http://sevahub.es), the design repository providing access to SEVA vectors converted to the Synthetic Biology Open Language (SBOL) (12) has been updated to the latest version of SynBioHub (46). This version adds new features (e.g. validation steps or improved SBOL-visual representation; 47) and bug fixes as outlined at https://github.com/SynBioHub/synbiohub/releases. Moreover, the new version lists the federated SynBioHub instances. The SEVA-to-SBOL conversion process has been improved as well: Previous SBOL files were generated manually by developing a software package to combine part sequences. In this update, we provide an automatic conversion tool that allows individual generation of SBOL encoded vectors and provides feedback on potential issues that may not initially be derived from the corresponding GenBank files (e.g. double annotations on the same sequence). Users can access this conversion tool at https://github.com/Biocomputation-CBGP/GBK2SBOL. Resulting SBOL files can then be used for the in-depth analysis of sequences and constructs (48) leading to advanced data integration strategies (49).

### Ordering SEVA plasmids

One of the trademarks of the SEVA platform is making standardized vectors freely available to all types of users, whether academic or industrial. All users have to do for ordering SEVA plasmids and Golden Standard vectors is filling the request form of the corresponding section of the SEVA webpage. Petitioners find there an easy procedure for asking (a reasonable number of) specific vectors. These are then shipped free of charge to individuals who contact through institutional email addresses of research centres, universities and accredited companies. For bulk orders of plasmids (10 or more), please contact the Webmaster as some handling fees could be applicable. Please note that we do not send materials requested through commercial email platforms or private addresses. Note also that we disclaim carrier liability. While most of the functional parts entered in the SEVA vectors enjoy freedom of operation, we cannot rule out that some given sequences are subject to some intellectual property (IP) rights. Should utilization of SEVA plasmids eventually led to a commercial application, users are encouraged to inspect the IP status of the corresponding DNA segments and deal with the issue with the original IP holder. In this way, IP and potential licensing issues are handed over to end-user scenarios rather than raising them upfront.

### Diffusion and communication: building a community of users

**Figure 4.**
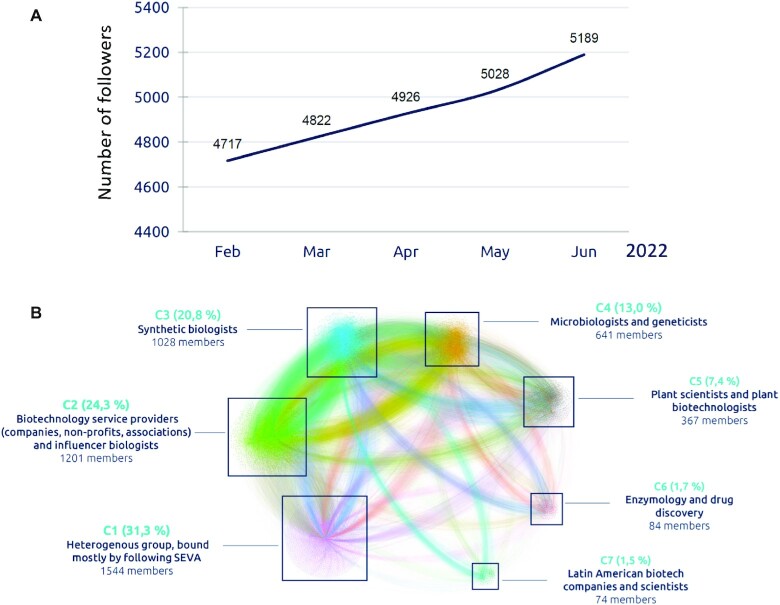
Mapping the community of SEVA users. The upper panel shows the steady increase of followers of the SEVA Twitter account https://twitter.com/SEVAplasmids in the first part of 2022. The profiles of all followers were downloaded through the Twitter API (application programming interface) and the resulting network of users visualized using Gephi software (https://gephi.org/) for complex node analysis. Each square represents a community of followers (nodes) with shared interests, language or geographic location (C1–C7), labelled with the percentage share of followers in that group. Denser clusters in the visualisation (such as C3 or C4) indicate closely-knit communities, wherein many users follow each other. Note predominance of synthetic biologists and biotechnology companies.

The setup of the first SEVA webpage in 2013 (10) was followed later (13) by the opening of a linked Twitter account (https://twitter.com/SEVAplasmids) along with an interactive blog with comments, novelties and posts related to vector development. More recently, these have been added with a free newsletter sent every three months to subscribers. External access to these platforms has enabled us to map and follow the evolution of the interest in the SEVA resource, especially in the period of time since the last update in 2020. As seen in Figure 1B, the number of website visits have remained basically stable during the last period (over 1000 per month), and the number of plasmids distributed (Figure 1C) kept over an average of 300 per year. Furthermore, adopting the number of followers in Twitter as a proxy of the interest and the professional allocation of users, allowed us to analyse and understand the communities created around the project as well as determining whether the platform reaches relevant audiences. The results (Figure 4) break down the field in seven distinct communities, the most differentiated being those self-described as Synthetic Biologists and Biotechnology Service providers. Beyond the number of citations of the SEVA platform since its first edition (>700 at the time of writing this article), these figures suggest an increased interest in the sort of genetic tools available through the site and they foreshow its further growth and demand. The same can be said for visits to the SEVA blog (https://seva-plasmids.com/blog/) and the quarterly Newsletter to which one can subscribe through the web page as well. Worth to mention also is the considerable visibility and appeal bestowed by the explanatory animation video (https://www.youtube.com/watch?v=I8gZjXDRQ2I) which appears in the SEVA homepage and provides a quick, user-friendly glimpse of what the platform is all about.

## OUTLOOK

What could be called the SEVA space includes a collection of general-purpose, broad host range cloning vectors, heterologous expression systems, reporters and specific purpose-constructs. The entries of the canonical vector collection, which is the core of the corpus, follows rigorously the arrangement of functional DNA segments established in the earliest publication (Figure 1A; 10) and which can be unequivocally cyphered according to a code (Figure 2) which in this case needs to be updated as the plasmid collection increases in number and utilities. Apart of the canonical set, the platform also includes a considerable number of constructs (SEVAsib) that capture part of the standard—but not all—without loss of functionality for specific purposes. These are also included in the repository and can be retrieved through the same channels and terms of the canonical collection.

Despite the number of excellent plasmid repositories available to molecular biologists and synthetic biologist, the distinguishing marks of the SEVA platform include (i) the emphasis on compositional standards for the sake of interoperability and reproducibility, (ii) the adoption of broad host range functionalities (antibiotic markers, replication origin, cargoes) and (iii) open and free upfront access to the materials at stake. While other repositories of biological materials may also take all or some of these features, we strongly advocate standardization as the way to go for moving the field of synthetic biology forward.

Since the last update of the SEVA DB we have noted publication of a number of articles that further develop the concept in different directions explicitly related to the original platform (50,51). In other cases, authors take on the overall SEVA view for the progress of their own developments in genetic tools but depart from the standard to different degrees and purposes (52–58). Finally, some conceptually independent proposals go also along the path of standardization of genetic assets without any explicit connection to the SEVA concept or format (59–63). It would be ideal that all these eventually converge in a single frame that eases genetic analyses and engineering of a large variety of microorganisms with compatible and comparable tools.

In the meantime, some additional expansion of the SEVA platform could be expected for improving e.g. the host range of the activities encoded in the constructs. This involves not just the replication origins but also expression signals for enabling genes to be expressed in a large variety of hosts (64). Also, there is considerable room for standardizing mini-transposon delivery vectors (and other integrative tools) beyond the prototypes currently listed in the SEVAsib Table. Finally, the growing trend to move from strain analysis and engineering to the same applied to microbiomes (65) asks also for novel, dedicated tools that are still in their infancy and deserve standardization efforts as well. These challenges will hopefully be met in subsequent updates of the platform.

## DATA AVAILABILITY

All relevant data is available through the SEVA platform (https://seva-plasmids.com). No new data were generated or analysed in support of this research.
